# Economic evaluation of germline genetic testing for breast cancer in low- and middle-income countries: a systematic review

**DOI:** 10.1186/s12885-024-12038-7

**Published:** 2024-03-07

**Authors:** Sook Pin Goh, Siew Chin Ong, Jue Ern Chan

**Affiliations:** 1 Discipline of Social and Administrative Pharmacy, School of Pharmaceutical Sciences, Universiti Sains Malaysia, Penang, Malaysia; 2 Pharmacy Department, Klinik Kesihatan Chemor Pejabat Kesihatan Daerah Kinta, Ipoh, Perak, Malaysia

**Keywords:** Genetic testing, Breast cancer, Systematic review, Economic evaluation, Low- and middle-income countries

## Abstract

**Background:**

Breast cancer (BC) is the most common cancer affecting women globally. Genetic testing serves as a prevention and treatment strategy for managing BC. This study aims to systematically review economic evaluations and the quality of selected studies involving genetic screening strategies for BC in low and middle-income countries (LMICs).

**Methods:**

A search was performed to identify related articles that were published up to April 2023 on PubMed, Embase, CINAHL, Web of Science, and the Centre for Reviews and Dissemination. Only English-language LMIC studies were included. Synthesis of studies characteristics, methodological and data input variations, incremental cost-effectiveness ratios (ICERs), and reporting quality (Consolidated Health Economic Evaluation Reporting Standards (CHEERS) 2022 checklist) were performed.

**Results:**

This review found five pertinent studies, mainly focusing on economic evaluations of germline genetic testing in upper-middle-income countries (Upper MICs) like Malaysia, China, and Brazil. Only one study covered multiple countries with varying incomes, including lower-middle-income nations (Lower MICs) like India. The ICERs values in various screening scenarios for early-stage BC, HER2 negative BC patients, and healthy women with clinical or family history criteria were ranging from USD 2214/QALY to USD 36,342/QALY. Multigene testing for all breast cancer patients with cascade testing was at USD 7729/QALY compared to BRCA alone. Most studies adhered to the CHEERS 2022 criteria, signifying high methodological quality.

**Conclusions:**

Germline testing could be considered as cost-effective compared to no testing in Upper MICs (e.g., Malaysia, China, Brazil) but not in Lower MICs (e.g., India) based on the willingness-to-pay (WTP) threshold set by each respective study. Limitations prevent a definite conclusion about cost-effectiveness across LMICs. More high-quality studies are crucial for informed decision-making and improved healthcare practices in these regions.

**Supplementary Information:**

The online version contains supplementary material available at 10.1186/s12885-024-12038-7.

## Background

Breast cancer (BC) is the most common cancer affecting women globally, with a staggering 2.26 million new cases documented in 2020 [1]. This remarkable figure establishes BC as the leading cancer in terms of new cases surpassing even lung cancer in prevalence across all genders and age groups. A positive sign of significant decline in mortality rates of BC by up to 40% in between 1989 and 2017 and with more than 90% of 5-year survival rates has been noted in few high income countries [2, 3]. However, these positive outcomes have not been observed in most of the low-income and middle-income countries (LMICs). This might be attributed to late-stage diagnosis or presentation and inadequate access to quality care which happened in sub-Saharan Africa [4, 5]. Therefore, highlighting the importance of early diagnosis and multimodality screening and treatment can help reduce mortality rates and improve survival rates of BC in LMICs.

About 5–10% of BC are considered to be due to an inheritable gene mutation [6]. Gaining insight into a patient’s genetic mutation status can aid in formulating a well-considered strategy for the management, treatment selection, and risk assessment of BC. For instance, an individual can choose a contralateral mastectomy after diagnosis with unilateral BC if patients carry cancer susceptibility genes (CSGs) [7, 8]. Relatives of BC patients carrying unaffected CSG can be identified via cascade testing and benefit from early diagnosis with frequent BC screening and surveillance, chemoprevention with an aromatase inhibitor or selective estrogen-receptor modulators (SERM) or surgical prevention such as risk-reducing mastectomy (RRM) [9–12]. Genetic testing analyzes an individual’s specific mutations or changes in a single gene [13]. With technological advancement, more efficient DNA-sequencing technologies are available. Multi-gene panel testing is often utilized as this panel includes testing other high-risk breast cancer genes besides from *BRCA1/2* gene only. Beyond the *BRCA1/2* gene, numerous new CSGs have emerged and been identified, such as *TP53, PTEN, CHEK2, ATM, and PALB2* [6]. In a recent study conducted by Yang et al. (2020), they discovered that PALB2 plays a significant role as one of the major cancer susceptibility genes [14]. The study reported that female BC patients might have an estimated BC risk of 53% (95% CI, 44–63%) up to the age of 80 years [14].

There were a few international guidelines such as the National Comprehensive Cancer Network (NCCN) [15] and the National Institute of Health and Care Excellence (NICE) [16] recommended genetic testing mainly for those with a family history or certain clinical criteria. Genetic testing is still limited in the Western health system even though the cost of genetic testing has decreased in recent years and several studies have shown the feasibility, practicability, and cost-effectiveness studies of genetic testing in BC [17]. This is far more limited and restricted access for LMICs. The prevalence of patients with the BRCA1/2 pathogenic variant without a strong family history of breast and/or ovarian cancer (OC) from LMICs such as China has accounted for up to 65.9% [18] compared to Western high-income countries with 15–50% [19, 20]. Hence, many studies suggested and supported the implementation of screening for all BC women and even for all healthy women [21–25]. LMICs are financially and resource-constrained despite the necessity of genetic testing for BC in LMICs. Therefore, a comprehensive systematic review of the existing evidence is needed to inform healthcare decision-making and resource allocation.

It is important to note that these studies have not comprehensively covered all the available genetic testing strategies despite multiple review studies had been attempting to summarize the economic evaluation of genetic testing for BC. For instance, D’Andrea et al. primarily focused on a single type of genetic testing, specifically the BRCA pathogenic variant [26]. Another systematic review conducted by Koldehoff and colleagues considered both BRCA and multigene testing but excluded studies involving population-based screening strategies and did not incorporate cascade testing [27]. Moreover, the scope of the reviews conducted by Koldehoff et al., Meshkani et al., and a review published in Chinese language were limited to studies published up to the year 2020 [27–29]. It is worth noting that few studies have been published after 2020, which have not been included in their reviews [27–29]. To the best of our knowledge, no review has been published to conclude and summarize the feasibility and practicality of genetic testing of BC in LMICs. The purpose of this study was to systematically review the variability and quality of existing economic evaluations of genetic testing for BC in LMIC settings by focusing on the following two specific objectives: (1) assess the different types of genetic screening strategies, and (2) synthesize and analyze the study characteristics and economic evidence of selected studies.

## Methods

### Study design

This systematic review was registered under International Prospective Register of Systematic Reviews (PROSPERO) with the registration number of CRD42023421284. The Preferred Reporting Items for Systematic Reviews and Meta-Analyses (PRISMA) guideline was employed for conducting and reporting in this review [30, 31].

### Search strategy

Five large electronic databases were searched: PubMed, EMBASE, Web of Science, Cumulative Index of Nursing and Allied Health Literature (CINAHL), and databases of the Centre for Reviews and Dissemination (CRD) including Database of Abstracts of Reviews of Effects (DARE), NHS Economic Evaluation Database (NHS EED) and Health Technology Assessment (HTA). Searches were conducted in May 2023 and were limited to articles published from its inception until April 2023. A manual search of the cited references and reference lists of included studies and systematic reviews was performed using Google Scholar in order to identify additional relevant studies. The systematic literature search was conducted using specific keywords such as “economic evaluation,” “genetic testing,” “breast cancer,” and “low- and middle-income country” to ensure a comprehensive exploration of the literature. The search strategy for all databases was summarized and presented in the Additional file 1. The search strategy was initially developed for PubMed and translated and applied to other databases afterward. The search included 137 LMICs as followed by the current classification income 2023 as listed from the database of World Bank [32].

### Eligibility criteria

The population (P), intervention (I), comparison (C), outcome (O), and study design (S) framework were used to identify relevant studies included in this systematic review study. The detailed exclusion and inclusion criteria are summarized in Table 1.

**Table 1:** Inclusion and exclusion criteria

	Inclusion criteria	Exclusion criteria
Population	All women, whether healthy with or without a family history or increased clinical risk of BC, as well as those already diagnosed with BC.	Male
Intervention	BRCA1/2 germline genetic testing and/or may include another genetic testing (i.e., pathogenic or likely pathogenic variants: BRCA1/2, PALB2, CHEK2, ATM, BARD1, RAD51c/d)	Somatic genetic testing, Single nucleotide polymorphism (SNP)
Comparison	No genetic testing or alternative screening methods	Not Applicable
Outcome	Costs per quality-adjusted life years (QALY), cost per life-years gained, and costs per number of cancer cases averted.	Cost analysis studies (e.g., with costs but no health outcomes)
Study Design	Partial or full economic evaluation such as cost-benefit analysis (CBA), cost-utility analysis (CUA) or/and cost-effectiveness analysis (CEA) as well as cost-minimization analysis (CMA). Studies conducted in randomized controlled trials, case studies, observational studies, or model-based studies were included.	No publication of full-text articles or original data such as systematic literature reviews, commentaries (letters to the editors, editorials), abstracts, and expert review
Language	English language	Other than the English language
Local setting	Low- and middle-income countries	High-income countries
Date of Publication	Until April 2023	May 2023 onwards

### Study selection process and data extraction

All the identified articles were first cross-checked and duplicates were removed. The remaining articles then underwent independent screening by two reviewers for titles and abstracts against the eligibility criteria. The eligible articles were then obtained in full-text format. Both reviewers independently screened and assessed the full texts to evaluate study eligibility. In cases where discrepancies or disagreements arose between the findings of the two reviewers, a discussion with a third reviewer was held to resolve.

A data extraction tool was developed and saved in Excel format. The information extracted from the articles included the author’s name, country of origin, type of genetic testing, study design, population, screening and treatment strategy, perspective, time horizon, discount rate, type of uncertainty analysis, and measured outcomes. All these data were subsequently presented and summarized in two separate tables.

### Data synthesis

To ensure comparability of the prices of genetic testing and incremental cost-effectiveness ratios (ICERs) across different currencies and years, the cost converter tool developed by the Campbell Collaboration (CC) and the Evidence for Policy and Practice Information and Coordinating Centre was employed. This valuable tool enabled the adjustment of values to international USD 2022 [33]. The cost converter tool, which is accessible online at no cost, employs the gross domestic product deflator index values and purchasing power parity conversion rates sourced from reputable organizations which as the International Monetary Fund and the Organization for Economic Co-operation and Development. It was not feasible to conduct a meta-analysis due to the substantial heterogeneity observed among the included studies regarding the screening target population.

### Assessment of quality of reporting

The quality assessment of the selected studies was conducted by employing the Consolidated Health Economic Evaluation Reporting Standards (CHEERS) 2022 statement. This updated statement consists of 28-item checklist with updated criteria that serve as a comprehensive guideline for transparent reporting and publication of health economic studies [34]. Identifiers of “yes”, “partial” and “no” were utilized to denote whether the reporting was “fully reported,” “partially reported,” or “not reported " when evaluating and assessing the selected articles. It is important to note that the CHEERS statement does not provide a specific scoring mechanism. Hence, identifiers were used to indicate the level of reporting in each study.

## Results

### Search results

**Fig. 1:**
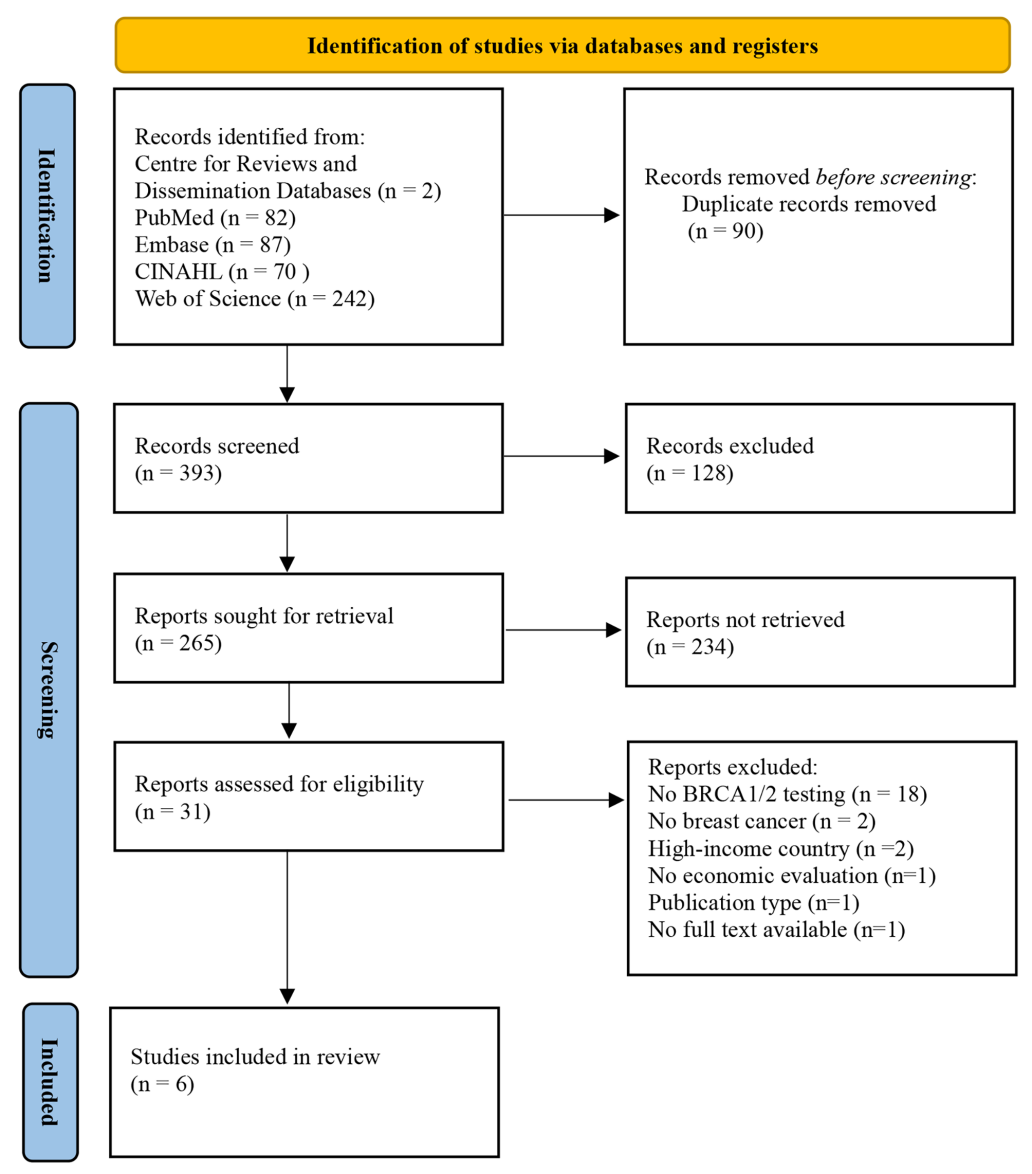
PRISMA 2020 flow diagram

The initial literature search yielded 483 articles, of which 90 duplicates were removed. A total of 393 articles were left for screening based on their titles. After excluding irrelevant titles, approximately 265 articles underwent further review. Screening based on abstracts further narrowed down the selection to 31 articles eligible for full-text screening. The main reasons for exclusion were the absence of economic study outcomes and the lack of genetic testing involvement in the studies. No additional relevant studies were found through the reference lists of the included studies. Ultimately, only six articles met all the inclusion criteria which allowed for data extraction and quality assessment [35–40]. Figure 1, the PRISMA 2020 flow diagram, provides a detailed summary of the selection process.

### Study characteristics

Table 2 demonstrates the key characteristics of the included studies [35–40]. The economic evaluations conducted in LMICs primarily originated from upper-middle-income countries (Upper MICs) like China (*n* = 2) Brazil (*n* = 2) and Malaysia (*n* = 1) [35, 36, 38–40] with only one study encompassed multiple countries with varying income levels. This study included multiple countries evaluating genetic testing in lower-middle-income countries (Lower MICs) such as India, in addition to high-income and upper-income countries [37]. All the studies were published after 2018 and employed both cost-utility and cost-effectiveness analysis, using the incremental cost-effectiveness ratio (ICER) as the outcome measure [35–40]. Half of the included articles (*n* = 3) considered a lifetime horizon [36–38] in the analysis of the studies. Only one study considered with the shortest time horizon being 20 years [35]. Out of the six studies, only two studies analyzed from both societal and payer perspectives [37, 38]. A discount rate of 3% was utilized for costs and effectiveness in more than half of the studies (*n* = 4) [35–38], while two studies applied a 5% discount rate specifically in the context of Brazil [39, 40]. All selected studies performed both deterministic and probabilistic sensitivity analysis [35–39].

**Table 2:** Characteristics of reviewed articles

Author (Year)	Country (country income category)	Population description	Treatment strategy	Intervention VS comparison	Study design	Perspective	Time horizon	Cascade Testing	discount rate	Type of uncertainty analysis
Genetic testing for breast cancer only										
Lim et al. (2018) [30]	Malaysia (UMIC)	Hypothetical cohort of 1000 patients who aged 40 years old with newly diagnosed as early stage (Stage1/2) unilateral BC.	risk-reducing mastectomy (RRM), risk-reducing bilateral salpingo-oophorectomy (RRBSO), tamoxifen chemoprevention, combination of these or neither	BRCA testing VS No testing, performed Routine clinical surveillance only	Decision tree and Markov Model (1 year length of cycle)	payer perspective	Lifetime	No	3% for costs and health outcomes	One way deterministic sensitivity analyses & probabilistic sensitivity analysis
Sun et al. (2022) [32]	China (UMIC)	All BC patients VS Family History/clinical-criteria-based testing	Prophylactic mastectomy and salpingo-oophorectomy	a)BRCA1/BRCA2/PALB2 testing for all BC patients b)BRCA1/BRCA2-testing for BC patients with FH/clinical criteria c) No testing	Microsimulation model at the individual level	Societal and Payer perspectives	Lifetime	Yes	3% for costs and health outcomes	One way deterministic sensitivity analyses & probabilistic sensitivity analysis
Wu et al. (2023) [29]	China (UMIC)	Patients with TNBC and hormone-receptor (HR)-positive and HER2-negative BC	Standard treatment with Olaparib and RRO as an adjuvant treatment	a) Universal gBRCAtesting for all TNBC and HR-positive HER2-negative BC patients b) No gBRCA testing c) Selected gBRCA testing	A decision tree analytic model based on transitional Markov Chain (1 year length of cycle)	Payer perspectives	20 years	No	3% for costs and health outcomes	One way deterministic sensitivity analyses & probabilistic sensitivity analysis
**Genetic testing for breast cancer and ovarian cancer**										
Manchanda et al. (2020) [31]	China (UMIC) & Brazil (UMIC) & India (LMIC)	Population-based screening for all women ≥ 30 years old.	RRSO, MMRI/mammography screening, chemoprevention with SERM, RRM	Population-based BRCA1/BRCA2 testing VS clinical-criteria/FH-based testing	Markov Model	Societal and Payer perspectives	Lifetime (China = 48 cycles; Brazil = 49 cycles; India = 38 cycles)	No	3% for costs and health outcomes	One way deterministic sensitivity analyses & probabilistic sensitivity analysis
Simoes Correa-Galendi et al. (2021) [33]	Brazil (UMIC)	Healthy women aged 30 years with personal or family history of BRCA-associated cancer and meeting the clinical criteria for genetic testing according to the National Comprehensive Cancer Network (NCCN).	Intensified surveillance, risk-reducing bilateral mastectomy and bilateral salpingo-oophorectomy	BRCA1/BRCA2 testing and counselling VS no genetic testing and counselling	Markov Model	Payer perspectives	70 years	No	5% for costs and utilities	One way deterministic sensitivity analyses & probabilistic sensitivity analysis
Lourencao et al. (2022) [34]	Brazil (UMIC)	Healthy women aged 30 years with personal or family history of BRCA-associated cancer and meeting the clinical criteria for genetic testing according to the National Comprehensive Cancer Network (NCCN).	Intensified surveillance, risk-reducing bilateral mastectomy, bilateral salpingo-oophorectomy, both bilateral mastectomy and bilateral salpingo-oophorectomy	BRCA1/BRCA2 testing and counselling and with surgical/non-surgical preventive options VS No genetic testing and counselling (with standard care)	Markov Model	Payer perspectives	70 years	Yes	5% for costs and utilities	Deterministic sensitivity analyses & probabilistic sensitivity analysis

### Summary of results from selected studies

Table 3 presents the results of all selected studies, including costs, parameters for outcome measurements, and ICERs [35–40].

**Table 3:** Results of economic evaluation of selected studies

Author (Year)	Source of cost data	International value of genetic testing (2022) USD	Source of effectiveness data	Willingness to pay threshold USD	Outcome measurement	Cost-effectiveness results, ICER* and conclusion from study
Genetic testing for breast cancer only						
Lim et al. (2018) [30]	Local Hospital	451.5 (2016) → 509.31 (2022)	Literature Search from Other Countries	9500 USD/QALY (1 time GDP per capita)	Incremental Costs per QALY (ICERs), Incremental Costs per LYS (ICERs)	• ICER: USD 2566/QALY; USD 918/life-year saved • Genetic testing is cost-effective compared to routine clinical surveillance as it was below WTP threshold.
Sun et al. (2022) [32]	Sampling database of the Chinese Urban Basic Medical Insurance	367 (2019) → 390.36 (2022)	Lifetime Tables from each country were obtained from the World Health Organization (WHO) and published literatures.	10,262 USD/QALY (1 time GDP per capita)	Incremental Costs per QALY (ICERs), Incremental Costs per LYG (ICERs)	• Multigene testing for all BC patients VS No genetic testing USD 4793/QALY, USD 4294/LYG (Societal) & USD 7729/QALY, USD 6923/LYG (payer perspective) • Unselected multigene testing to all BC patients in China is cost-effective as compared with no testing or selected testing as it was below WTP threshold
Wu et al. (2023) [29]	Price Announcement by the Shanghai Health Minister of China	308.6 (2021) → 314.98 (2022)	Published literatures	31,500 USD /QALY	**Primary Outcome**: QALY gained & ICERs **Secondary Outcome**: life expectancy gained & survival outcome	• Universal gBRCA testing among TNBC patient compared with no testing and selected testing respectively, ICERs of USD 10,812/QALY and USD 11,218 /QALY • Universal testing for all HER2-negative BC patients compared with no testing and selected testing respectively, with ICERs of USD 2214/QALY & USD 2065/QALY. • Universal gBRCA testing is cost-effective as the ICER value is below the WTP threshold.
**Genetic Testing for Breast Cancer and Ovarian Cancer**						
Manchanda et al. (2020) [31]	*China* Urban Basic Medical Insurance Database; *Brazil* Management System of Procedures/Medical drugs/Orthotics/Prosthetics/Special Materials (SIGTAP), the Health Price Bank (BPS), and Chamber of Regulation of the Market of Medicines (CMED); *India* Accredited Cancer Centre Tata Medical Centre	200 (2016) → 225.61 (2022)	Lifetime Tables from each country were obtained from the World Health Organization (WHO); QALY values obtained from published literatures.	China: ($15,531/QALY-$46,592/QALY) Brazil: ($15,182/QALY-$45,545/QALY India: ($6574/QALY-$19,722/QALY) WTP were based on 1–3 times GDP of each country	Lifetime costs and QALYs, ICERs	• **ICER** *China* Societal: USD 26,716/LY, USD 20,379/QALY Payer: USD 34,730/LY, USD 26,492/QALY *Brazil* Societal: USD 17,873/LY, USD 15,318/QALY Payer: USD 27,632/LY, USD 23,683/QALY *India* Societal: USD 31,831/LY, USD 25,980/QALY Payer: USD 44,527/LY, USD 36,342/QALY • BRCA testing is cost-effective in both China and Brazil except India from both payer and societal perspective when compared to the WTP threshold set in the study
Simoes Correa-Galendi et al. (2021) [33]	Official Brazilian Universal Health Coverage System (SUS) database and local distributors	1480 (2019) → 1574.22 (2022)	Several systematic literature searches in Medline and BIREME (a Latin American health database)	Not well defined	Incremental Costs per QALY (ICERs), Incremental Costs per LYG (ICERs)	• ICER: USD 12,472/QALY & USD 14,013/ LYG • Cost-effectiveness of BRCA testing is still depends on undecided cost-effectiveness threshold. The ICER is 1.04 times the GDP per capita
Lourencao et al. (2022) [34]	Official Brazilian Universal Health Coverage System (SUS) database	524.98 (2021)→ 535.84 (2022)	Published studies from a systematic literature search in the PubMed database	R$ 25,000/QALY (U$ 11, 563.37/QALY) Based on the lowest thresholds reported in National Commission for the Incorporation of Technologies (CONITEC)	Incremental Costs per QALY (ICERs), Incremental Costs per LYG (ICERs)	• ICER: USD 5618/QALY and USD 5188/ LYG • BRCA testing is cost-effective if the WTP is USD 11,563.37/QALY

Breast Cancer (BC); Quality-adjusted life years (QALY); Incremental cost-effectiveness ratios (ICERs); life-year gained (LYG); Triple-negative breast cancer (TNBC); Gross Domestic Product (GDP); Willingness-to Pay (WTP)

*ICER: Converted to USD 2022, using the CCEMG-EPPI-Centre Cost Converter [33]

### Genetic screening and treatment strategies (population, age, and intervention and comparison groups)

The selected studies examined various target populations. The age groups for conducting BC genetic screening were clearly defined in most of the studies [36, 37, 39, 40] except for two studies [35, 38]. Most of the studies (*n* = 3) that included the defined age groups suggested a minimum screening age of 30 years old [37, 39, 40] in their analysis. Interestingly, only one study included women from the general population who were aged 30 years or older [37]. Conversely, the majority of the studies (*n* = 5) focused on particular conditions such as BC patients or individuals meeting clinical criteria or having a family history of the disease [35, 36, 38–40]. Five out of six studies concentrated exclusively on BRCA1/2 genetic testing [35–37, 39, 40], while only one study involved multigene testing including BRCA1, BRCA2 and PALB2 [38]. Additionally, only one study conducted cascade multigene testing for individuals who received positive test results [38]. Women who tested positive in the screening group were provided with risk-reducing mastectomy (RRM), risk-reducing salpingo-oophorectomy (RRSO), or both as treatment options [35–40] in all the six studies. Only one study went a step further by offering standard treatment along with a Poly-(ADP)-ribose polymerase (PARP) inhibitor, specifically Olaparib as a treatment strategy for those who tested positive [35]. There were only two studies that addressed the risk of developing coronary heart disease (CHD) in women who had performed RRSO [37, 38], while the remaining studies did not take into account of the potential negative effects associated with RRSO [35, 36, 39, 40].

### Variation in methodological approaches and data inputs

Table 3 presents the cost estimation approaches and sources of effectiveness data utilized in the selected studies [35–40]. The majority of cost data were derived or estimated from local data sources, including local hospitals [36, 37], the National Health System [35, 39, 40], and Medical Insurance Databases [37, 38]. As for effectiveness data, the most prevalent approach for all 6 studies [35–40] was to obtain utility values from previously published literature studies while prioritizing data from local studies if possible.

There were notable variations in the uptake rate of risk reducing options such as RRM and/or RRSO and/or chemoprevention. All the assessed studies reported the uptake rate of RRM alone, ranging from 7 to 47% for unaffected individuals [37–40] and 21–53.9% for affected individuals [35–38]. On the other hand, the uptake rate of RRSO alone ranged from 16.8 to 55% for unaffected individuals [37–40] and 13.3–60% for affected individuals [35–38]. There were only three studies that considered the inclusion of chemoprevention options, such as tamoxifen, with an uptake rate ranging from 7 to 16.3%, aiming to reduce the risk of breast cancer [36–38]. Among the studies, only Lim et al. considered separate input data for the uptake rate of risk reducing options in the context of having one or two remaining breast [36]. However, Lim et al. assumed that patients made decisions for risk-reducing treatments within a year after testing positive and those who opted for RRM removed all remaining breasts [36]. Moreover, half of the assessed studies did not explicitly mention in the context about the assumptions for age at which patients adopted risk reduction surgeries [35, 36, 40]. Two studies assumed the median ages for RRM and RRSO in unaffected BRCA carriers were 37 and 40 years old respectively based on previous studies and data [37, 38]. Only one study that categorized the adoption rates of prophylactic surgeries by age groups found that women aged 35–39 years old had the highest rate of RRM at 11.2% and RRSO at 27.4% for those aged 30–34 years old [39, 40]. It was noticed that the adoption rate of RRSO almost doubled when compared to RRM across all age categories [39]. Two studies employed local data [36, 40] whereas four studies applied data obtained from published articles from other countries [35, 37–39].

Data on the effectiveness of preventive surgeries were heterogeneous across the studies. The models in all the assessed studies indicated that varying the input of the effectiveness of preventive surgeries in sensitivity analyses changed the ICER values, ranging from 20 to 48% [39], nearly doubling [40] the ICER values observed in the base case analysis. The hazard ratio (HR) for the development of BC after RRM alone varied, ranging from 6 to 9% for unaffected carriers [37–39] and 18–48% for those diagnosed with BC [38, 39]. Meanwhile, RRSO alone demonstrated a significant reduction up to 98.6% in unaffected carriers [40] and 65% in those diagnosed with BC [38] for the occurrence of ipsilateral or contralateral breast cancer. Moreover, Sun et al. was the only study reported the HR for BC survival from RRSO (46%) and contralateral prophylactic mastectomy (37%) in addition to the risk reduction in developing BC and/or OC after risk reducing surgeries [38]. Sun et al. also incorporated the HR for BC survival from contralateral prophylactic mastectomy and RRSO in the one-way sensitivity analyses [38]. BRCA1/2-positive patients were considered for various potential risk-reducing surgeries. However, individuals diagnosed solely with PALB2 positive were not included in RRSO and OC risk reduction analyses [38]. If included, it might further enhance the ICER values. The important point to emphasize was that no study accounted for the survival benefits of the testing itself as it was perceived the benefit was contingent upon the risk-reducing treatments.

Utility value is one of the important input parameters for cost-utility analyses. All the studies [35–38, 40] incorporated utility data into the sensitivity analyses of the model, except for one study [39]. Only two studies [39, 40] offered information about the methods used to combine utility data in which employing the multiplicative method, while others did not specify the method employed [35–38]. The changes in the utility data were applied to variables including positive or negative tests [39], prophylactic or treatment measures for positive results [35–40], cancer states [35–40], and post-cancer states [40].

Most of the studies (*n* = 4) didn’t take into account of percentage of error such as false positive and false negative results [37–40]. However, Lim et al. and Wu et al. assumed a high positive predictive value of 99–100% and a negative predictive value of 93–99%, as they believed that the sensitivity and specificity of the tests used were high [35, 36].

Concerning the decision analytical models employed, the Markov model was the most frequently utilized, either as a standalone model in three studies [37, 39, 40]or in combination with decision tree modeling in two studies [35, 36]. Only one study incorporated a microsimulation model at the individual level [38]. Moreover, it is noteworthy that only four out of the included studies (67%) reported the utilization of model validation methods [36, 38–40]. These three studies conducted evaluations of face validity [36, 38–40] and technical validity [38–40] to assess the reliability of their models. Furthermore, two studies incorporated external validation, specifically employing cross-model validation techniques [39, 40]. Additionally, only one study assessed the validation status of the model using the Assessment of the Validation Status of Health-Economic Decision Models (AdViSHE) framework providing further robustness to their findings [39].

### Analysis of the price of genetic testing and incremental cost-effectiveness ratios (ICERs)

Table 3 provides information on the unadjusted and adjusted prices of genetic testing, as well as the outcome measurements of adjusted ICERs in the selected studies [35–40]. The majority of studies (5 out of 6) reported adjusted prices of genetic testing below USD 550 [35–38, 40], except for one study that reported a cost of USD 1574.22 for BRCA genetic testing [39]. All studies presented outcomes in both quality-adjusted life year (QALY) and life-year gained (LYG) [35–40].

The ICER values obtained were used to compare against a willingness-to-pay (WTP) threshold in all the selected studies (*n* = 6) [35–40]. All the six included studies concluded the genetic testing in their study were cost-effective as the results showed were less than the WTP threshold employed in the studies [35–40]. The WTP thresholds in the three studies [36–38] were clearly defined, and one study [35] was assumed threshold based on the recommendations by the World Health Organization-CHOICE (1–3 times GDP per capita). However, two articles focusing on the Brazil setting adopted more conservative thresholds (R$ 25,000) as recommended in the reports of the National Commission for the Incorporation of Technologies (CONITEC), which presented three levels of thresholds [39, 40]. Both BRCA genetic testing and multigene testing (including BRCA1/BRCA2/PALB2) were found to dominate the no testing or testing limiting to family history or clinical criteria in Upper MICs such as Malaysia [36], China [35, 37, 38], and Brazil [37, 40] from payer perspective or both payer and societal perspectives. From the payer perspective, screening of BRCA testing among early-stage BC, HER2 negative BC patients, healthy women with clinical or family history criteria, and population-based screening for all healthy women was cost-effective when compared with the WTP thresholds set by the authors which was within the range of ICER USD 2214/QALY to USD 36,342/QALY [35–37, 39, 40]. Compared to BRCA alone, multigene testing for all breast cancer patients with cascade testing was USD 7729/QALY [38]. However, BRCA testing was not deemed cost-effective for population-based screening in all women aged 30 years or older in Lower MIC like India from both payer and societal perspectives as the ICERs values were USD 36,342/QALY and USD 25,980/QALY respectively [37].

### Assessment of quality of selected studies

Overall, the selected studies demonstrated satisfactory quality and included the majority of the essential elements, as outlined in Table 4. However, most studies (*n* = 5) did not sufficiently clarify the interventions being compared in the title [35, 37–40], except Lim et al. (2018). Additionally, nearly all of the selected studies did not or partially incorporate approaches or effects related to engaging or involving patients or other stakeholders affected by the study (CHEERS 2022 criteria 21 and 24) [35–40].

**Table 4:** CHEERS 2022 checklist quality results

Item	CHEERS Criteria	Authors (Year)					
		Lim et al. (2018) [30]	Sun et al. (2022) [32]	Wu et al. (2023) [29]	Manchanda et al. (2020) [31]	Simoes Correa-Galendi et al. (2021) [33]	Lourencao et al. (2022) [34]
**Title**							
1	Title	Yes	Partial	Partial	Partial	Partial	Partial
**Abstract**							
2	Abstract	Yes	Partial	Yes	Partial	Yes	Partial
**Introduction**							
3	Background and Objectives	Yes	Yes	Yes	Yes	Yes	Yes
**Methods**							
4	Health economic analysis plan	Yes	Yes	Yes	Yes	Yes	Yes
5	Study population	Yes	Yes	Yes	Yes	Yes	Yes
6	Setting and location	Yes	Yes	Yes	Yes	Yes	Yes
7	Comparators	Yes	Yes	Yes	Yes	Yes	Yes
8	Perspective	Yes	Yes	Partial	Yes	Yes	Yes
9	Time horizon	Partial	Partial	Partial	Yes	Yes	Yes
10	Discount rate	Yes	Yes	Yes	Yes	Yes	Yes
11	Selection of outcomes	Yes	Yes	Yes	Yes	Yes	Yes
12	Measurement of outcomes	Yes	Yes	Yes	Yes	Yes	Yes
13	Valuation of outcomes	Yes	Yes	Yes	Yes	Yes	Partial
14	Measurement and valuation of resources and costs	Yes	Yes	Yes	Yes	Yes	Yes
15	Currency, price date, and conversion	Yes	Yes	Yes	Yes	Yes	Yes
16	Rationale and description of model	Yes	Yes	Yes	Partial	Yes	Partial
17	Analytics and assumptions	Partial	Partial	Yes	Yes	Yes	Yes
18	Characterizing heterogeneity	Yes	Yes	No	Yes	Yes	Yes
19	Characterizing distributional effects	Yes	Yes	Yes	Yes	Yes	Yes
20	Characterizing uncertainty	Partial	Partial	Yes	Partial	Partial	Partial
21	Approach to engagement with patients and others affected by the study	Partial	Partial	Partial	No	No	Partial
**Results**							
22	Study parameters	Yes	Yes	Yes	Yes	Yes	Yes
23	Summary of main results	Yes	Yes	Yes	Yes	Yes	Yes
24	Effect of uncertainty	Partial	Partial	Yes	Partial	Partial	Yes
25	Effect of engagement with patients and others affected by the study	No	No	No	No	No	No
**Discussion**							
26	Study findings, limitations, generalizability, and current knowledge	Yes	Yes	Yes	Yes	Yes	Yes
**Other relevant information**							
27	Source of funding	Yes	Yes	Yes	Yes	Yes	Yes
28	Conflicts of interest	Yes	Yes	Yes	Yes	Yes	Yes

Note Fully Reported: Yes, Partially Reported: Partial, Not Reported: No

One positive aspect was that all of the studies included in the review presented thorough and transparent information regarding their findings, limitations, sources of funding, and potential conflicts of interest [35–40]. This ensured that crucial aspects of the research were fully reported.

## Discussion

The present systematic review identified six relevant full-text studies published in LMICs after 2018 which focused on the economic evaluation of genetic testing for BC. Interestingly, all of these studies were published within the past five years [35–40] despite our search period encompassing articles before April 2023. This might be attributed to the increasing recognition of the importance of genetic testing in both the prevention and treatment of BC [41, 42] as well as the decreasing costs associated with such testing [43, 44]. Furthermore, there are increasing evidence demonstrating the cost-effectiveness of genetic testing implementation in developed countries might have contributed to the recent surge in research on this related topic [17, 44].

To the best of our knowledge, this article was the first systematic review for the economic evaluation of genetic testing of BC in LMICs. Plenty of articles included in this review are conducted in Upper MICs only [35, 36, 38–40]. Hence, it is important to note that the findings may not be directly applicable to Lower MICs and low-income countries. Manchanda et al. evaluated population-based BRCA testing in multiple countries such as high-income countries as well as Upper and Lower MICs [37]. They determined that the testing was cost-effective in high and Upper MICs from both the payer and societal perspectives. However, the test was not deemed cost-effective from either perspective unless the cost of BRCA testing was reduced to below $172 per test, making it cost-effective from a societal perspective for Lower MICs such as India [37]. Therefore, caution should be exercised when generalizing the outcomes of the reviewed studies to LMICs, particularly those with lower income levels.

The utilization of multigene testing has gained significant traction with the introduction of advanced technologies such as Next-Generation Sequencing (NGS) [45]. This innovative approach enables the analysis of multiple genes, offering advantages such as reduced costs, shorter turnaround times, and greater benefits when compared to limited BRCA1/2 testing [46]. Notably, China is currently the sole Upper MIC that has conducted a cost-effectiveness evaluation of multigene testing for BC and the ICER value is below the WTP threshold (one-times GDP per capita) [38]. In addition to the BRCA1/2 genes, several other pathogenic variants with moderate-to-high penetrance, including ATM, BARD1, CHEK2, PALB2, PTEN, and TP53, have been identified as genes associated with an increased risk of BC [47]. Research conducted by Li and colleagues further supported the cost-effectiveness of multigene testing for screening and treatment which demonstrating improvements in life expectancy for women with a family history of BC [48]. However, it is crucial to consider the potential risks associated with multigene testing, such as the detection of Variants of Unknown Significance (VUS) and gene variants with unclear clinical implications [49, 50]. Policymakers can consider tailoring BC screening and therapeutic strategies based on the results of multigene testing which takes into account the cumulative lifetime risk of BC (high/moderate/low) and thus optimizing patient care and more cost-effective. Brunei serves as a notable example of recognizing the significance of personalized care. Brunei set a screening policy with an extended mammography screening interval of every 3 years starting at the age of 40. However, Brunei also prioritizes women at high genetic risk, specifically those carrying BRCA1/2 mutations. For this selected group, annual screening is recommended, commencing as early as 25 years of age [51].

In many LMICs, the current practice revolves around clinical or family-history-based criteria and a 10% BRCA risk threshold probability [16] when considering genetic testing. However, there is a concern about the underutilization of genetic testing as some eligible patients might not be referred for testing [19, 52]. This limited access and utilization of genetic testing result in many high-risk women missing out on the opportunity for early detection and better treatment options for their BC. While a few studies have proven the cost-effectiveness of population-based BRCA testing specifically among Ashkenazi Jews [53, 54] and mainly from the United Kingdom and United States payers’ perspectives [17], it is important to recognize that a one-size-fits-all policy cannot be implemented. Among the included studies in our review, Manchanda et al. [37] was the only article that assessed the cost-effectiveness of population-based screening for all healthy women, showing it to be cost-effective in high-income countries and Upper MICs but not Lower MICs. On the other hand, the remaining five studies focused on clinical and family history-based criteria, comparing them to no testing [35, 36, 38–40]. These studies demonstrated the cost-effectiveness of genetic testing among those with early-stage BC [36], all HER2 negative cases [35], all BC women [38], as well as healthy women with clinical criteria and family history [39, 40] in LMICs. Based on these findings, it is necessary to consider factors such as population characteristics, availablility of the resources, and regional differences when assessing the applicability and feasibility of the study in a country and consequently making policy amendments. Simplified guidelines and tailored strategies should be developed to improve access to and utilization of genetic testing in order to ensure that high-risk women in LMICs have the opportunity to obtain early detection and appropriate treatment options for BC. It is crucial to address the potential impact of complex guidelines and any instances of malpractice among physicians as these factors can influence the implementation of current genetic screening policies [55]. Nonetheless, it is crucial to take into account the ethical, legal, and social consequences of introducing population-based genetic screening programs.

Markov model, either alone or in combination with the decision tree model, are commonly used in the included studies from LMICs as decision analytic models [35–37, 39, 40]. The decision tree model is mainly applied when recurring events are not important and the nature of events mainly occurs over a short period. In contrast, Markov model simulates a lot of different health states over time. On the other hand, the microsimulation model offers greater flexibility in capturing event timing and interdependencies which in turn provides a more nuanced representation of real-world dynamics [56, 57]. The microsimulation model is particularly well-suited for cascade testing analysis as the interaction between individuals is important as well as able to incorporate individual-specific factors such as age, health state, disease progression, treatment response, and adherence to interventions. Out of the included studies, only one conducted by Sun et al. utilized a microsimulation model at the individual level to assess the economic outcomes of multigene testing in BC patients which including the testing of first and second-degree relatives [38]. A review published by Zischke et al. also supported the adoption of more sophisticated modeling techniques such as discrete event simulation or dynamic simulation models as it can incorporate patient heterogeneity and varying patient pathways [58]. Earlier research indicated that studies utilizing basic decision trees may overstate the advantages by neglecting to consider competing risks over time [59]. However, the choice between these models depends on the specific research question, the availability of data, the desired level of detail, and also the accuracy of the analysis.

Another crucial aspect to consider is the uptake rate of preventive strategies, such as RRM and RRSO as well as the potential adverse effects of these strategies for individuals who tested positive for genetic mutations. The uptake rates of these surgeries are sensitive parameters that can introduce uncertainty in the outcomes of economic modeling studies [27]. According to the data from an international database encompassing 10 countries, the rate of RRM was highest in the United States (50%) and lowest in Poland (4.5%), while the uptake of RRSO was highest in France (83%) and lowest in China (37%) [60]. Therefore, the heterogeneity in uptake rates may, to some extent be explained by cultural differences across countries [27] and hence making it challenging to generalize findings. Health economic modeling studies should consider the potential impact of age-related differences in uptake rates on the incremental cost-effectiveness ratios (ICER) of these preventive strategies [61]. Petelin et al. also supported this notion by demonstrating that the rates of adherence to the screening program among individuals with BRCA pathogenic variants increase notably with age. As a result, the outcomes would be different for women who enroll in the program at later stages of life [62]. It is also crucial to account for the potential for delaying preventive surgeries or opting for intensive surveillance as alternative strategies. Notably, Simoes Correa-Galendi et al. was one of the few studies that accounted for these possibilities in their analysis [39]. Moreover, there have been conflicting findings regarding the effects of RRSO on BC risk in individuals with BRCA mutations, and concerns have been raised about potential adverse consequences including fertility loss, premature menopause, and psychological and physical challenges [11, 63]. It is worth noting that the study conducted by Wu et al. did not consider the negative impact or adverse outcomes associated with risk-reducing surgeries (RRSO and RRM) and the use of PARP inhibitors like Olaparib, which is an adjuvant treatment for high-risk HER2 negative patients with positive BRCA testing [35]. In contrast, Sun et al. [38] and Manchanda et al. [37] showed a good example by considering the potential negative effects of RRSO such as an increased risk of developing CHD.

The reporting quality was assessed using the latest version of the CHEERS 2022 checklist [34] which provides comprehensive guidance for transparent reporting in health economic studies. However, it was observed that all the identified studies [35–40] did not fully address the criteria newly added checklist items, especially regarding the approach and impact of engaging with patients and other stakeholders affected by the study. Inclusive engagement with all relevant parties such as patients, communities, the public, and stakeholders like clinicians or payers is essential to ensure the validity and relevance of the study findings. Several studies have shown that involving stakeholders as research partners can have a significant influence on study protocols and even the outcomes [64, 65]. Teppala et al. suggested future research should consider the patients’ and stakeholders’ preferences when assessing the cost-effectiveness of germline testing in comparison to other healthcare priorities in their studies [66]. Furthermore, it is crucial for future research to incorporate such engagement strategies and prioritize the perspectives of patients and stakeholders in the evaluation of genetic testing interventions.

In contrast to the systematic reviews conducted by D’Andrea et al. [26] and Koldehoff et al. [27], our study encompasses a broader range of genetic testing strategies for BC, including but not limited to BRCA testing, population-based screening, and included those studies without cascade testing. Moreover, we have included the additional three studies from LMICs compared to the previous review by Meshkani et al. [28], who did not impose geographical restrictions on their study selection. The evidence compiled in our review further reinforces the cost-effectiveness of genetic testing in LMICs by incorporating more recent studies published after 2020. Our findings aligned with those reviewed by D’Andrea et al. [26] and Meshkani et al. [28] in highlighting the significance of genetic testing prices as a crucial factor in determining the cost-effectiveness of BC genetic testing especially in Lower MICs such as India [37]. While our included studies mainly analyze women aged over 30 years [37, 39, 40], it is worth noting that the review summarized by Koldehoff et al. [27] found that most of the included studies focused on women aged 40 years as the preferred age group. Similar to our review, there were no studies in their analysis that provided sensitivity analysis for different age groups. Meshkani et al. concluded that cascade genetic testing is a cost-effective strategy [28], but our study could not provide a conclusive verdict on the cost-effectiveness of cascade testing in LMICs. This limitation arose from the fact that only one study included in our analysis incorporated cascade testing in their model analysis [38]. Researchers must consider the number of tested relatives in their sensitivity analyses as highlighted by Zischke et al. as this factor could significantly influence the overall costs [58]. However, Sun et al. did not conduct such sensitivity analyses in their studies [38]. Therefore, further research and evidence are required to have a better understanding of the economic implications of cascade testing in LMIC settings.

### Strengths and limitations

This systematic review represented a pioneering effort to consolidate the latest evidence about the economic evaluations of genetic testing for BC screening in LMICs. This study not only summarized the latest findings but also assessed study quality, methodologies, and identified research gaps for future exploration. Nevertheless, the limitations of this review still exist. Firstly, the restriction to English-language articles might lead to the exclusion of valuable publications in other languages which might potentially be limiting the comprehensiveness of the review. Secondly, this review excluded the grey literature might have minimal impact on the overall findings, as the quality assessment of grey literature is often challenging. Thirdly, it is worth noting that the costs and benefits associated with BRCA genetic testing were inherently taken into account factors related to OC, as BRCA mutations are known to be associated with an increased risk of developing OC. Many of the excluded studies utilized similar modeling approaches with those studies only focused on breast cancer. Although this review did not specifically include studies focusing on OC, the exclusion of OC models did not significantly alter the overall conclusions drawn from this review.

### Future research

To address the existing gaps, it is crucial to conduct rigorous economic evaluation studies on genetic testing in LMICs. These studies should focus on the economic assessments of how multigene testing can be advantageous for countries like those with Lower MICs and nations with limited resources, different healthcare settings, and different populations. Adherence to economic guidelines like the CHEERS 2022 checklist is important to enhance research transparency and reliability which enables standardized reporting for better comparability. Real-world data such as patient-reported and clinical outcomes as well as economic data can provide insights into practical settings. This approach assists in the decision-making on coverage and reimbursement policies. The need for real-world data (RWD) evidence is urgently needed especially in oncology, to evaluate the effectiveness of personalized medicine comprehensively [67].

## Conclusion

This study revealed that germline genetic testing is cost-effective when compared to no testing in Upper MICs like Malaysia, China, and Brazil based on the WTP threshold set by each respective study. However, genetic testing made its implementation less favorable in Lower MICs such as India due to the higher cost. This study found germline genetic testing showed positive economic value in various scenarios including early-stage BC, all BC patients, HER2 negative cases, as well as healthy individuals with clinical or family histories. Although there’s growing interest in personalized care, economic assessments of genetic testing in LMICs remain lacking. Due to diverse interventions and populations, the study couldn’t conclusively establish cost-effectiveness for BC genetic testing across all LMICs. Despite limited evidence, the study provides valuable insights and anticipates wider accessibility of genetic testing as costs decrease and technology advances nowadays. High-quality economic evaluation studies in LMICs are needed to enhance healthcare decision-making.

### Electronic supplementary material

Below is the link to the electronic supplementary material.

**Additional file 1:** Search Strategy and Results; Rationale for excluded studies in systematic review

## Data Availability

All data generated or analysed during this study are included in this published article and its supplementary files/additional files.

## Abbreviations

BC: breast cancer
LMICs: low-income and middle-income countries
CSGs: cancer susceptibility genes
RRM: risk-reducing mastectomy
SERM: selective estrogen-receptor modulators
NCCN: National Comprehensive Cancer Network
NICE: National Institute of Health and Care Excellence
PROSPERO: International Prospective Register of Systematic Reviews
PRISMA: Preferred Reporting Items for Systematic Reviews and Meta-Analyses
CINAHL: Cumulative Index of Nursing and Allied Health Literature
CRD: Centre for Reviews and Dissemination
DARE: Database of Abstracts of Reviews of Effects
NHS EED: NHS Economic Evaluation Database
HTA: Health Technology Assessment
ICERs: incremental cost-effectiveness ratios
CC: Campbell Collaboration
CHEERS: Consolidated Health Economic Evaluation Reporting Standards
Upper MICs: upper-middle-income countries
Lower MICs: lower-middle-income countries
RRSO: risk-reducing salpingo-oophorectomy
PARP: Poly-(ADP)-ribose polymerase
CHD: coronary heart disease
AdViSHE: Assessment of the Validation Status of Health-Economic Decision Models
LYG: Life-year gained
QALY: Quality-adjusted life year
WTP: willingness-to-pay
GDP: gross domestic product
OC: ovarian cancer
HR: hazard ratio
