# Supplementary material for: Economic evaluation of germline genetic testing for breast cancer in low- and middle-income countries: a systematic review
Source: BMC Cancer. 2024 Mar 7;24:316. doi: 10.1186/s12885-024-12038-7 (PMC10919043; doi:10.1186/s12885-024-12038-7)
Supplement: Supplementary file 1 — Additional file 1: Search Strategy and Results; Rationale for excluded studies in systematic review [file 12885_2024_12038_MOESM1_ESM.docx]

**Supplementary material**

**Article title**

Economic Evaluation of Germline Genetic Testing for Breast Cancer in Low- and Middle-Income Countries: A Systematic Review

**Journal name**

BMC Cancer

**Authors**

Sook Pin Goh, Siew Chin Ong*, Jue Ern Chan

*Corresponding author

Email: [siewchinong@usm.my](mailto:siewchinong@usm.my)

Discipline of Social and Administrative Pharmacy, School of Pharmaceutical Sciences, Universiti Sains

Malaysia, Penang, Malaysia

**Funding**

This study was funded by the Malaysian Ministry of Higher Education, Fundamental Research Grant Scheme (grant no: FRGS/1/2022/SS06/USM/02/8).

**Conflict of Interests**

Sook Pin Goh, Siew Chin Ong, and Jue Ern Chan declare that they have no conflict of interests.

The search terms were formulated according to the population, intervention, comparator, outcome, and study (PICOS) framework. The search approach utilized Boolean operators like "OR" to link search terms within each domain, and the "AND" Boolean operator between different PICOS domains.

**Table S1: General Search Terms**

**Table S2: Search Results of PUBMED**

**Table S3: Search Results of EMBASE**

**Table S4: Search Results of Web of Science (Dated: 14/5/2023)**

**Table S5: Search Results of CINAHL (dated: 14/5/2023)**

**Table S6: Search Results of Databases of the Centre for Reviews and Dissemination (CRD) including Database of Abstracts of Reviews of Effects (DARE), NHS Economic Evaluation Database (NHS EED) and Health Technology Assessment (HTA)**

**Table S7: Rationale for excluded studies in systematic review**

**Table S1: General Search Terms**

| Economic Evaluation | Cost analysis  Cost Effectiveness  Cost Benefit  Cost Utility  Economic Evaluation  Health Economic |
| --- | --- |
| Genetic testing | Genetic test  Germline test  Gene test  DNA test  Genetic screen  BRCA1/2,  PALB2,  CHEK2,  ATM,  BARD1,  RAD51c/d |
| Breast Cancer | breast neoplasms  breast cancer |
| LMICs | Afghanistan  Angola  Albania  Argentina  Armenia  American Samoa  Azerbaijan  Burundi  Benin  Burkina Faso  Bangladesh  Bulgaria  Bosnia and Herzegovina  Belarus  Belize  Bolivia  Brazil  Bhutan  Botswana  Central African Republic  China  Côte d’Ivoire  Cameroon  Congo, Dem. Rep.  Congo, Rep.  Colombia  Comoros  Cabo Verde  Costa Rica  Cuba  Djibouti  Dominica  Dominican Republic  Algeria  Ecuador  Egypt, Arab Rep.  Eritrea  Ethiopia  Fiji  Micronesia, Fed. Sts.  Gabon  Georgia  Ghana  Guinea  Gambia, The  Guinea-Bissau  Equatorial Guinea  Grenada  Guatemala  Guyana  Honduras  Haiti  Indonesia  India  Iran, Islamic Rep.  Iraq  Jamaica  Jordan  Kazakhstan  Kenya  Kyrgyz Republic  Cambodia  Kiribati  Lao PDR  Lebanon  Liberia  Libya  St. Lucia  Sri Lanka  Lesotho  Morocco  Moldova  Madagascar  Maldives  Mexico  Marshall Islands  North Macedonia  Mali  Myanmar  Montenegro  Mongolia  Mozambique  Mauritania  Mauritius  Malawi  Malaysia  Namibia  Niger  Nigeria  Nicaragua  Nepal  Pakistan  Peru  Philippines  Palau  Papua New Guinea  Korea, Dem. People's Rep.  Paraguay  West Bank and Gaza  Russian Federation  Rwanda  Sudan  Senegal  Solomon Islands  Sierra Leone  El Salvador  Somalia  Serbia  South Sudan  São Tomé and Príncipe  Suriname  Eswatini  Syrian Arab Republic  Chad  Togo  Thailand  Tajikistan  Turkmenistan  Timor-Leste  Tonga  Tunisia  Türkiye  Tuvalu  Tanzania  Uganda  Ukraine  Uzbekistan  St. Vincent and the Grenadines  Venezuela, RB  Vietnam  Vanuatu  Samoa  Kosovo  Yemen, Rep.  South Africa  Zambia  Zimbabwe |

**Table S2: Search Results of PUBMED (dated: 13/5/2023)**

| **Set Number** | **Search Term for Pubmed** | **Results** |
| --- | --- | --- |
| #1 Genetic testing | "genetic testing"[MeSH Terms] OR "genetic testing"[MeSH Terms] OR "Germline"[Title/Abstract] OR "dna"[MeSH Terms] OR "genes"[MeSH Terms] OR "BRCA1"[Title/Abstract] OR "BRCA2"[Title/Abstract] OR "PALB2"[Title/Abstract] OR "CHEK2"[Title/Abstract] OR "RAD51c"[Title/Abstract] OR "RAD51d"[Title/Abstract] OR "ATM"[Title/Abstract] OR "BARD1"[Title/Abstract] | 1,555,108 |
| #2 Breast Cancer | "breast neoplasms"[MeSH Terms] OR ("breast"[All Fields] AND "neoplasms"[All Fields]) OR "breast neoplasms"[All Fields] OR ("breast neoplasms"[MeSH Terms] OR ("breast"[All Fields] AND "neoplasms"[All Fields]) OR "breast neoplasms"[All Fields] OR ("breast"[All Fields] AND "cancer"[All Fields]) OR "breast cancer"[All Fields]) | 491,729 |
| #3 Low and middle income country | "developing countries"[MeSH Terms] OR ("developing"[All Fields] AND "countries"[All Fields]) OR "developing countries"[All Fields] OR ("developing"[All Fields] AND "country"[All Fields]) OR "developing country"[All Fields] OR "under-developed"[Title/Abstract] OR "underdeveloped"[Title/Abstract] OR "lmic"[Title/Abstract] OR "low resource"[Title/Abstract] OR "under-resourced"[Title/Abstract] OR "resource poor"[Title/Abstract] OR ("low"[Title/Abstract] AND "middle"[Title/Abstract] AND "income"[Title/Abstract]) OR "Afghanistan"[Title/Abstract] OR "Angola"[Title/Abstract] OR "Albania"[Title/Abstract] OR "Argentina"[Title/Abstract] OR "Armenia"[Title/Abstract] OR "american samoa"[Title/Abstract] OR "Azerbaijan"[Title/Abstract] OR "Burundi"[Title/Abstract] OR "Benin"[Title/Abstract] OR "burkina faso"[Title/Abstract] OR "Bangladesh"[Title/Abstract] OR "Bulgaria"[Title/Abstract] OR ("Bosnia"[Title/Abstract] AND "Herzegovina"[Title/Abstract]) OR "Belarus"[Title/Abstract] OR "Belize"[Title/Abstract] OR "Bolivia"[Title/Abstract] OR "Brazil"[Title/Abstract] OR "Bhutan"[Title/Abstract] OR "Botswana"[Title/Abstract] OR "central african republic"[Title/Abstract] OR "China"[Title/Abstract] OR "cote d ivoire"[Title/Abstract] OR "Cameroon"[Title/Abstract] OR (("congo"[MeSH Terms] OR "congo"[All Fields]) AND "dem rep"[Title/Abstract]) OR (("congo"[MeSH Terms] OR "congo"[All Fields]) AND "rep"[Title/Abstract]) OR "Colombia"[Title/Abstract] OR "Comoros"[Title/Abstract] OR "cabo verde"[Title/Abstract] OR "costa rica"[Title/Abstract] OR "Cuba"[Title/Abstract] OR "Djibouti"[Title/Abstract] OR "Dominica"[Title/Abstract] OR "dominican republic"[Title/Abstract] OR "Algeria"[Title/Abstract] OR "Ecuador"[Title/Abstract] OR ((("egypt"[MeSH Terms] OR "egypt"[All Fields] OR "egypt s"[All Fields]) AND ("arabs"[MeSH Terms] OR "arabs"[All Fields] OR "Arab"[All Fields])) AND "rep"[Title/Abstract]) OR "Eritrea"[Title/Abstract] OR "Ethiopia"[Title/Abstract] OR "Fiji"[Title/Abstract] OR ((("micronesia"[MeSH Terms] OR "micronesia"[All Fields]) AND "fed"[All Fields]) AND "sts"[Title/Abstract]) OR "Gabon"[Title/Abstract] OR "Georgia"[Title/Abstract] OR "Ghana"[Title/Abstract] OR "Guinea"[Title/Abstract] OR "gambia the"[Title/Abstract] OR "Guinea-Bissau"[Title/Abstract] OR "equatorial guinea"[Title/Abstract] OR "Grenada"[Title/Abstract] OR "Guatemala"[Title/Abstract] OR "Guyana"[Title/Abstract] OR "Honduras"[Title/Abstract] OR "Haiti"[Title/Abstract] OR "Indonesia"[Title/Abstract] OR "India"[Title/Abstract] OR ((("iran"[MeSH Terms] OR "iran"[All Fields]) AND ("islam"[MeSH Terms] OR "islam"[All Fields] OR "islamic"[All Fields] OR "islam s"[All Fields] OR "islamism"[All Fields])) AND "rep"[Title/Abstract]) OR "Iraq"[Title/Abstract] OR "Jamaica"[Title/Abstract] OR "Jordan"[Title/Abstract] OR "Kazakhstan"[Title/Abstract] OR "Kenya"[Title/Abstract] OR "kyrgyz republic"[Title/Abstract] OR "Cambodia"[Title/Abstract] OR "Kiribati"[Title/Abstract] OR "lao pdr"[Title/Abstract] OR "Lebanon"[Title/Abstract] OR "Liberia"[Title/Abstract] OR "Libya"[Title/Abstract] OR "st lucia"[Title/Abstract] OR "sri lanka"[Title/Abstract] OR "Lesotho"[Title/Abstract] OR "Morocco"[Title/Abstract] OR "Moldova"[Title/Abstract] OR "Madagascar"[Title/Abstract] OR "Maldives"[Title/Abstract] OR "Mexico"[Title/Abstract] OR "marshall islands"[Title/Abstract] OR "north macedonia"[Title/Abstract] OR "Mali"[Title/Abstract] OR "Myanmar"[Title/Abstract] OR "Montenegro"[Title/Abstract] OR "Mongolia"[Title/Abstract] OR "Mozambique"[Title/Abstract] OR "Mauritania"[Title/Abstract] OR "Mauritius"[Title/Abstract] OR "Malawi"[Title/Abstract] OR "Malaysia"[Title/Abstract] OR "Namibia"[Title/Abstract] OR "Niger"[Title/Abstract] OR "Nigeria"[Title/Abstract] OR "Nicaragua"[Title/Abstract] OR "Nepal"[Title/Abstract] OR "Pakistan"[Title/Abstract] OR "Peru"[Title/Abstract] OR "Philippines"[Title/Abstract] OR "Palau"[Title/Abstract] OR "papua new guinea"[Title/Abstract] OR ((("korea"[MeSH Terms] OR "korea"[All Fields] OR "korea s"[All Fields] OR "koreas"[All Fields]) AND "dem"[All Fields] AND ("people s"[All Fields] OR "peopled"[All Fields] OR "peopling"[All Fields] OR "persons"[MeSH Terms] OR "persons"[All Fields] OR "people"[All Fields] OR "peoples"[All Fields])) AND "rep"[Title/Abstract]) OR "Paraguay"[Title/Abstract] OR ("west bank"[Title/Abstract] AND "Gaza"[Title/Abstract]) OR "russian federation"[Title/Abstract] OR "Rwanda"[Title/Abstract] OR "Sudan"[Title/Abstract] OR "Senegal"[Title/Abstract] OR "solomon islands"[Title/Abstract] OR "sierra leone"[Title/Abstract] OR "el salvador"[Title/Abstract] OR "Somalia"[Title/Abstract] OR "Serbia"[Title/Abstract] OR "south sudan"[Title/Abstract] OR ("sao tome"[Title/Abstract] AND "Principe"[Title/Abstract]) OR "Suriname"[Title/Abstract] OR "Eswatini"[Title/Abstract] OR "syrian arab republic"[Title/Abstract] OR "Chad"[Title/Abstract] OR "Togo"[Title/Abstract] OR "Thailand"[Title/Abstract] OR "Tajikistan"[Title/Abstract] OR "Turkmenistan"[Title/Abstract] OR "Timor-Leste"[Title/Abstract] OR "Tonga"[Title/Abstract] OR "Tunisia"[Title/Abstract] OR "Turkiye"[Title/Abstract] OR "Tuvalu"[Title/Abstract] OR "Tanzania"[Title/Abstract] OR "Uganda"[Title/Abstract] OR "Ukraine"[Title/Abstract] OR "Uzbekistan"[Title/Abstract] OR ("st vincent"[Title/Abstract] AND "the grenadines"[Title/Abstract]) OR (("venezuela"[MeSH Terms] OR "venezuela"[All Fields] OR "venezuela s"[All Fields]) AND "RB"[Title/Abstract]) OR "Vietnam"[Title/Abstract] OR "Vanuatu"[Title/Abstract] OR "Samoa"[Title/Abstract] OR "Kosovo"[Title/Abstract] OR (("yemen"[MeSH Terms] OR "yemen"[All Fields]) AND "rep"[Title/Abstract]) OR "south africa"[Title/Abstract] OR "Zambia"[Title/Abstract] OR "Zimbabwe"[Title/Abstract] | 1,357,389 |
| #4 STEP 1 | #1 AND #2 AND #3 | 941 |
| #5 Economic Evaluation | "costs and cost analysis"[MeSH Terms] OR "cost effectiveness analysis"[MeSH Terms] OR "cost benefit analysis"[MeSH Terms] OR "health care economics and organizations"[MeSH Terms] OR ("cost benefit analysis"[MeSH Terms] OR ("cost benefit"[All Fields] AND "analysis"[All Fields]) OR "cost benefit analysis"[All Fields] OR ("economic"[All Fields] AND "evaluation"[All Fields]) OR "economic evaluation"[All Fields]) OR "cost"[Title/Abstract] | 2,090,316 |
| #6 STEP 2 | #4 AND #5 | 82 |

**Table S3: Search Results of EMBASE (dated: 14/5/2023)**

| **Set Number** | **Search Term for EMBASE** | **Results** |
| --- | --- | --- |
| #1 | 'genetic testing'/exp OR Germline;ab,ti OR Genetic*;ab,ti OR dna;ab,ti OR genes;ab,ti OR BRCA1;ab,ti OR BRCA2;ab,ti OR PALB2;ab,ti OR CHEK2;ab,ti OR RAD51c;ab,ti OR RAD51d;ab,ti OR ATM;ab,ti OR BARD1;ab,ti | 134,356 |
| #2 | ‘breast cancer’/exp OR ‘breast neoplasm’/exp OR ‘breast tumor’/exp OR ‘breast cancer’:ab,ti OR ‘breast neoplasm’:ab,ti OR ‘breast tumor’:ab,ti | 716,421 |
| #3 | ‘developing countries’/exp OR ‘under-developed’:ab,ti OR ‘underdeveloped’:ab,ti OR ‘lmic’:ab,ti OR ‘low resource’:ab,ti OR ‘under-resourced’:ab,ti OR ‘resource poor’:ab,ti OR (‘low’:ab,ti AND ‘middle’:ab,ti AND ‘income’:ab,ti) OR ‘Afghanistan’:ab,ti OR ‘Angola’:ab,ti OR ‘Albania’:ab,ti OR ‘Argentina’:ab,ti OR ‘Armenia’:ab,ti OR ‘american samoa’:ab,ti OR ‘Azerbaijan’:ab,ti OR ‘Burundi’:ab,ti OR ‘Benin’:ab,ti OR ‘burkina faso’:ab,ti OR ‘Bangladesh’:ab,ti OR ‘Bulgaria’:ab,ti OR (‘Bosnia’:ab,ti AND ‘Herzegovina’:ab,ti) OR ‘Belarus’:ab,ti OR ‘Belize’:ab,ti OR ‘Bolivia’:ab,ti OR ‘Brazil’:ab,ti OR ‘Bhutan’:ab,ti OR ‘Botswana’:ab,ti OR ‘central african republic’:ab,ti OR ‘China’:ab,ti OR ‘cote d ivoire’:ab,ti OR ‘Cameroon’:ab,ti OR ‘Congo, Dem rep’:ab,ti OR ‘Congo, Rep’:ab,ti OR ‘Colombia’:ab,ti OR ‘Comoros’:ab,ti OR ‘cabo verde’:ab,ti OR ‘costa rica’:ab,ti OR ‘Cuba’:ab,ti OR ‘Djibouti’:ab,ti OR ‘Dominica’:ab,ti OR ‘dominican republic’:ab,ti OR ‘Algeria’:ab,ti OR ‘Ecuador’:ab,ti OR ‘Egypt Arab Rep.’:ab,ti OR ‘Eritrea’:ab,ti OR ‘Ethiopia’:ab,ti OR ‘Fiji’:ab,ti OR ‘micronesia’:ab,ti OR ‘Gabon’:ab,ti OR ‘Georgia’:ab,ti OR ‘Ghana’:ab,ti OR ‘Guinea’:ab,ti OR ‘gambia’:ab,ti OR ‘Guinea-Bissau’:ab,ti OR ‘equatorial guinea’:ab,ti OR ‘Grenada’:ab,ti OR ‘Guatemala’:ab,ti OR ‘Guyana’:ab,ti OR ‘Honduras’:ab,ti OR ‘Haiti’:ab,ti OR ‘Indonesia’:ab,ti OR ‘India’:ab,ti OR ‘Iran’:ab,ti OR ‘Iraq’:ab,ti OR ‘Jamaica’:ab,ti OR ‘Jordan’:ab,ti OR ‘Kazakhstan’:ab,ti OR ‘Kenya’:ab,ti OR ‘kyrgyz republic’:ab,ti OR ‘Cambodia’:ab,ti OR ‘Kiribati’:ab,ti OR ‘lao pdr’:ab,ti OR ‘Lebanon’:ab,ti OR ‘Liberia’:ab,ti OR ‘Libya’:ab,ti OR ‘st lucia’:ab,ti OR ‘sri lanka’:ab,ti OR ‘Lesotho’:ab,ti OR ‘Morocco’:ab,ti OR ‘Moldova’:ab,ti OR ‘Madagascar’:ab,ti OR ‘Maldives’:ab,ti OR ‘Mexico’:ab,ti OR ‘marshall islands’:ab,ti OR ‘north macedonia’:ab,ti OR ‘Mali’:ab,ti OR ‘Myanmar’:ab,ti OR ‘Montenegro’:ab,ti OR ‘Mongolia’:ab,ti OR ‘Mozambique’:ab,ti OR ‘Mauritania’:ab,ti OR ‘Mauritius’:ab,ti OR ‘Malawi’:ab,ti OR ‘Malaysia’:ab,ti OR ‘Namibia’:ab,ti OR ‘Niger’:ab,ti OR ‘Nigeria’:ab,ti OR ‘Nicaragua’:ab,ti OR ‘Nepal’:ab,ti OR ‘Pakistan’:ab,ti OR ‘Peru’:ab,ti OR ‘Philippines’:ab,ti OR ‘Palau’:ab,ti OR ‘papua new guinea’:ab,ti OR ‘Democratic People Republic of Korea’:ab,ti OR ‘Paraguay’:ab,ti OR ‘West bank and Gaza’:ab,ti OR ‘russian federation’:ab,ti OR ‘Rwanda’:ab,ti OR ‘Sudan’:ab,ti OR ‘Senegal’:ab,ti OR ‘solomon islands’:ab,ti OR ‘sierra leone’:ab,ti OR ‘el salvador’:ab,ti OR ‘Somalia’:ab,ti OR ‘Serbia’:ab,ti OR ‘south sudan’:ab,ti OR ‘sao tome and principe’:ab,ti OR ‘Suriname’:ab,ti OR ‘Eswatini’:ab,ti OR ‘syrian arab republic’:ab,ti OR ‘Chad’:ab,ti OR ‘Togo’:ab,ti OR ‘Thailand’:ab,ti OR ‘Tajikistan’:ab,ti OR ‘Turkmenistan’:ab,ti OR ‘Timor-Leste’:ab,ti OR ‘Tonga’:ab,ti OR ‘Tunisia’:ab,ti OR ‘Turkiye’:ab,ti OR ‘Tuvalu’:ab,ti OR ‘Tanzania’:ab,ti OR ‘Uganda’:ab,ti OR ‘Ukraine’:ab,ti OR ‘Uzbekistan’:ab,ti OR ‘st Vincent and the grenadines’:ab,ti OR ‘Venezuela’:ab,ti OR ‘Vietnam’:ab,ti OR ‘Vanuatu’:ab,ti OR ‘Samoa’:ab,ti OR ‘Kosovo’:ab,ti OR ‘Republic of Yemen’:ab,ti OR ‘south africa’:ab,ti OR ‘Zambia’:ab,ti OR ‘Zimbabwe’:ab,ti | 1,677,981 |
| #4 STEP 1 | #1 AND #2 AND #3 | 452 |
| #5 | ‘costs and cost analysis’/exp OR ‘cost effectiveness analysis’/exp OR ‘cost benefit analysis’/exp OR ‘health economic’/exp OR ‘cost utility’/exp OR ‘economic evaluation’/exp OR ‘cost’:ab,ti OR ‘cost effectiveness analysis’:ab,ti OR ‘cost benefit analysis’:ab,ti OR ‘health economic’:ab,ti OR ‘cost utility’:ab,ti OR ‘economic evaluation’:ab,ti | 1,092,996 |
| #6 STEP 2 | #4 AND #5 | 87 |


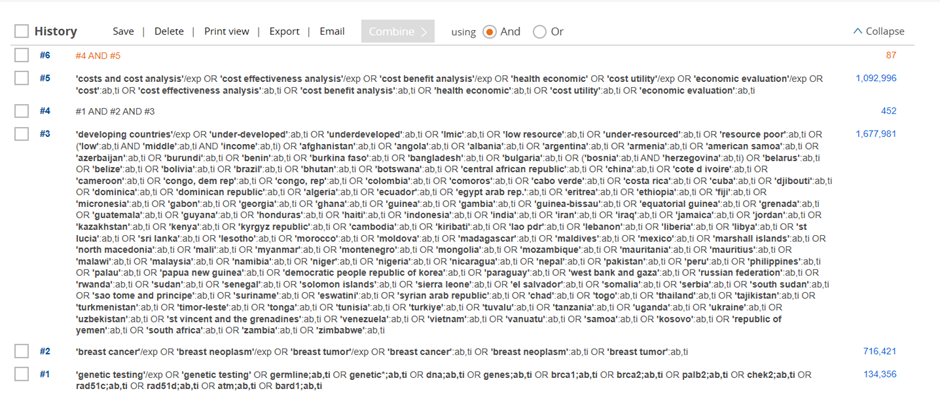


**Table S4: Search Results of Web of Science (Dated: 14/5/2023)**

| **Set Number** | **Search Term for Web of Science** | **Results** |
| --- | --- | --- |
| #1 | ((((((((((((ALL=(genetic testing)) OR ALL=(germline)) OR ALL=(genetic* screening)) OR ALL=(DNA)) OR ALL=(gene)) OR AB=(BRCA1)) OR AB=(BRCA2)) OR AB=(PALB2)) OR AB=(CHEK2)) OR AB=(RAD51c)) OR AB=(RAD51d)) OR AB=(ATM)) OR AB=(BARD1) | 4,688,124 |
| #2 | ((ALL=(breast cancer)) OR ALL=(breast tumo?r)) OR ALL=(breast neoplasm) | 707,427 |
| #3 | (((((((((((((((((((((((((((((((((((((((((((((((((((((((((((((((((((((((((((((((((((((((((((((((((((((((((((((((((((((((((((((((((((((((((((((((ALL=(developing countries)) OR ALL=(underdeveloped)) OR ALL=(under developed)) OR AB=(LMIC)) OR AB=(low resource)) OR AB=(under-resourced)) OR AB=(resource poor)) OR AB=(low and middle income)) OR AB=(Afghanistan)) OR AB=(Angola)) OR AB=(Albania)) OR AB=(Argentina)) OR AB=(Armenia)) OR AB=(American Samoa)) OR AB=(Azerbaijan)) OR AB=(Burundi)) OR AB=(Benin)) OR AB=(Burkina Faso)) OR AB=(Bangladesh)) OR AB=(Bulgaria)) OR AB=(Bosnia and Herzegovina)) OR AB=(Belarus)) OR AB=(Belize)) OR AB=(Bolivia)) OR AB=(Brazil)) OR AB=(Bhutan)) OR AB=(Botswana)) OR AB=(Central African Republic)) OR AB=(China)) OR AB=(Côte d’Ivoire)) OR AB=(Cameroon)) OR AB=(Congo)) OR AB=(Colombia)) OR AB=(Comoros)) OR AB=(Cabo Verde)) OR AB=(Costa Rica)) OR AB=(Cuba)) OR AB=(Djibouti)) OR AB=(Dominica)) OR AB=(Dominican Republic)) OR AB=(Algeria)) OR AB=(Ecuador)) OR AB=(Egypt)) OR AB=(Eritrea)) OR AB=(Ethiopia)) OR AB=(Fiji)) OR AB=(Micronesia)) OR AB=(Gabon)) OR AB=(Georgia)) OR AB=(Ghana)) OR AB=(Guinea)) OR AB=(Gambia)) OR AB=(Guinea-Bissau)) OR AB=(Equatorial Guinea)) OR AB=(Grenada)) OR AB=(Guatemala)) OR AB=(Guyana)) OR AB=(Honduras)) OR AB=(Haiti)) OR AB=(Indonesia)) OR AB=(India)) OR AB=(Iran)) OR AB=(Iraq)) OR AB=(Jamaica)) OR AB=(Jordan)) OR AB=(Kazakhstan)) OR AB=(Kenya)) OR AB=(Kyrgyz Republic)) OR AB=(Cambodia)) OR AB=(Kiribati)) OR AB=(Lao PDR)) OR AB=(Lebanon)) OR AB=(Liberia)) OR AB=(Libya)) OR AB=(St. Lucia)) OR AB=(Sri Lanka)) OR AB=(Lesotho)) OR AB=(Morocco)) OR AB=(Moldova)) OR AB=(Madagascar)) OR AB=(Maldives)) OR AB=(Mexico)) OR AB=(Marshall Islands)) OR AB=(North Macedonia)) OR AB=(Mali)) OR AB=(Myanmar)) OR AB=(Montenegro)) OR AB=(Mongolia)) OR AB=(Mozambique)) OR AB=(Mauritania)) OR AB=(Mauritius)) OR AB=(Malawi)) OR AB=(Malaysia)) OR AB=(Namibia)) OR AB=(Niger)) OR AB=(Nigeria)) OR AB=(Nicaragua)) OR AB=(Nepal)) OR AB=(Pakistan)) OR AB=(Peru)) OR AB=(Philippines)) OR AB=(Palau)) OR AB=(Papua New Guinea)) OR AB=( Democratic People’s Republic of Korea)) OR AB=(Paraguay)) OR AB=(West Bank and Gaza)) OR AB=(Russian Federation)) OR AB=(Rwanda)) OR AB=(Sudan Senegal)) OR AB=(Solomon Islands)) OR AB=(Sierra Leone)) OR AB=(El Salvador)) OR AB=(Somalia)) OR AB=(Serbia)) OR AB=(South Sudan)) OR AB=(São Tomé and Príncipe)) OR AB=(Suriname)) OR AB=(Eswatini)) OR AB=(Syrian Arab Republic)) OR AB=(Chad)) OR AB=(Togo)) OR AB=(Thailand)) OR AB=(Tajikistan)) OR AB=(Turkmenistan)) OR AB=(Timor-Leste)) OR AB=(Tonga)) OR AB=(Tunisia)) OR AB=(Türkiye)) OR AB=(Tuvalu)) OR AB=(Tanzania)) OR AB=(Uganda)) OR AB=(Ukraine)) OR AB=(Uzbekistan)) OR AB=(St. Vincent and the Grenadines)) OR AB=(Venezuela)) OR AB=(Vietnam)) OR AB=(Vanuatu)) OR AB=(Samoa)) OR AB=(Kosovo)) OR AB=(Yemen)) OR AB=(South Africa)) OR AB=(Zambia)) OR AB=(Zimbabwe)) | 3,769,026 |
| #4 STEP 1 | #1 AND #2 AND #3 | 5,571 |
| #5 | (((((ALL=(economic evaluation)) OR ALL=(cost effectiveness)) OR ALL=(cost benefit)) OR ALL=(cost utility)) OR ALL=(health economic)) OR ALL=(cost analysis) | 1,181,315 |
| #6 STEP 2 | #4 AND #5 | 242 |

**Table S5: Search Results of CINAHL (dated: 14/5/2023)**

| **Set Number** | **Search Term for Web of Science** | **Results** |
| --- | --- | --- |
| S1 | ( genetic testing or screening ) OR gene OR TX dna testing OR TX germline OR AB BARD1 OR AB BRCA1 OR AB BRCA2 OR AB PALB2 OR AB CHEK2 OR AB RAD51c OR AB RAD51d OR AB ATM | 442,659 |
| S2 | breast cancer OR breast neoplasm OR breast tumo#r | 124,209 |
| S3 | developing countries OR underdeveloped OR under developed OR LMIC OR low resource OR under-resource OR resource poor OR ( low and middle income ) | 82,350 |
| S4 | AB Afghanistan OR AB Angola OR AB Albania OR AB Argentina OR AB Armenia OR AB American Samoa OR AB Azerbaijan OR AB Burundi OR AB Benin OR AB Burkina Faso OR AB Bangladesh  OR AB Bulgaria OR AB Bosnia and Herzegovina OR AB Belarus  OR AB Belize OR AB Bolivia OR AB Brazil OR AB Bhutan OR AB Botswana OR AB Central African Republic OR AB China OR AB Côte d’Ivoire OR AB Cameroon OR AB Congo OR AB Colombia OR AB Comoros OR AB Cabo Verde OR AB Costa Rica OR AB Cuba OR AB Djibouti OR AB Dominica OR AB Dominican Republic OR AB Algeria OR AB Ecuador OR AB Egypt OR AB Eritrea OR AB Ethiopia OR AB Fiji OR AB Micronesia OR AB Gabon OR AB Georgia OR AB Ghana OR AB Guinea OR AB Gambia OR AB Guinea-Bissau OR AB Equatorial Guinea OR AB Grenada OR AB Guatemala OR AB Guyana OR AB Honduras OR AB Haiti OR AB Indonesia OR AB India OR AB Iran OR AB Iraq OR AB Jamaica OR AB Jordan OR AB Kazakhstan OR AB Kenya OR AB Kyrgyz Republic OR AB Cambodia OR AB Kiribati OR AB Lao PDR OR AB Lebanon OR AB Liberia OR AB Libya OR AB St. Lucia OR AB Sri Lanka OR AB Lesotho OR AB Morocco OR AB Moldova OR AB Madagascar OR AB Maldives OR AB Mexico OR AB Marshall Islands OR AB North Macedonia OR AB Mali OR AB Myanmar OR AB Montenegro OR AB Mongolia OR AB Mozambique OR AB Mauritania OR AB Mauritius OR AB Malawi OR AB Malaysia OR AB Namibia OR AB Niger OR AB Nigeria OR AB Nicaragua OR AB Nepal OR AB Pakistan OR AB Peru OR AB Philippines OR AB Palau OR AB Papua New Guinea OR AB Democratic People’s Republic of Korea OR AB Paraguay OR AB West Bank and Gaza OR AB Russian Federation OR AB Rwanda OR AB Sudan OR AB Senegal OR AB Solomon Islands OR AB Sierra Leone OR AB El Salvador OR AB Somalia OR AB Serbia OR AB South Sudan OR AB São Tomé and Príncipe OR AB Suriname OR AB Eswatini OR AB Syrian Arab Republic OR AB Chad OR AB Togo OR AB Thailand OR AB Tajikistan OR AB Turkmenistan OR AB Timor-Leste OR AB Tonga OR AB Tunisia OR AB Türkiye OR AB Tuvalu  OR AB Tanzania OR AB Uganda OR AB Ukraine OR AB Uzbekistan OR AB St. Vincent and the Grenadines OR AB Venezuela OR AB Vietnam OR AB Vanuatu OR AB Samoa OR AB Kosovo OR AB Yemen OR AB South Africa OR AB Zambia OR AB Zimbabwe | 180,360 |
| S5 | S3 OR S4 | 207,079 |
| S6 | TX economic evaluation OR TX cost effectiveness OR TX cost benefit OR TX cost utility OR TX cost analysis OR TX health economic | 167,050 |
| S7 | S1 AND S2 AND S5 AND S6 | 70 |

**Table S6: Search Results of Databases of the Centre for Reviews and Dissemination (CRD) including Database of Abstracts of Reviews of Effects (DARE), NHS Economic Evaluation Database (NHS EED) and Health Technology Assessment (HTA)**


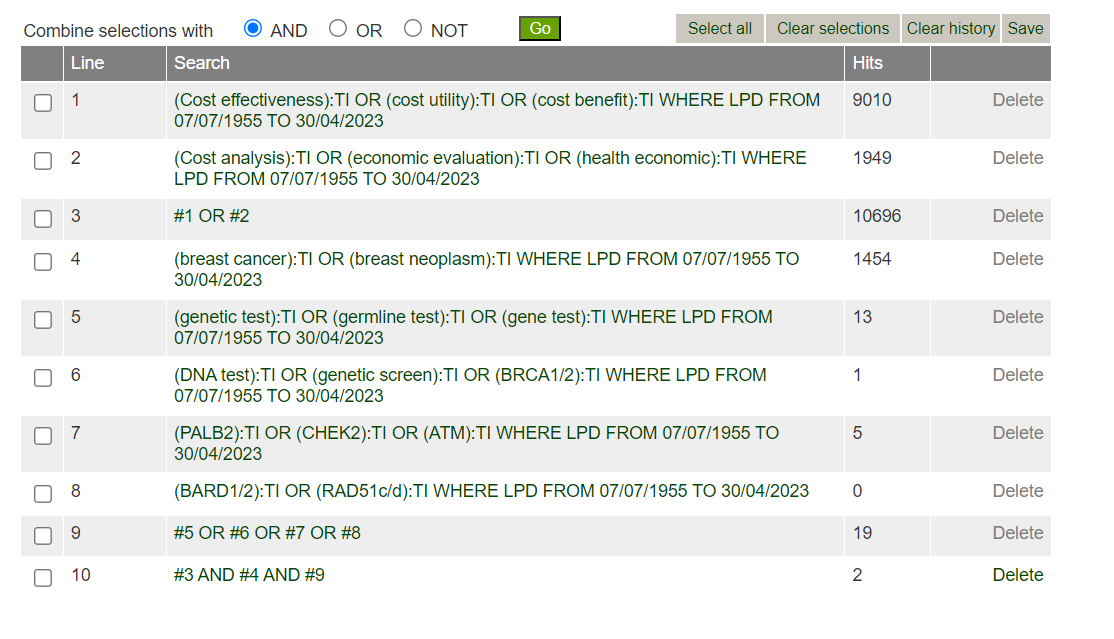


**Table S7: Rationale for excluded studies in systematic review**

| **Study** | **Reason** |
| --- | --- |
| Lim, K., Yoon, S., Teo, S., Taib, N. M., Shabaruddin, F., Dahlui, M., & Chaiyakunapruk, N. (2016). Cost Effectiveness of Brca Mutation Testing for Early-Stage Breast Cancer Patients with Family History in Malaysia. Value in health, 19(7), A889. | Conference abstract, No full text available |
| Vekov, T., & Dzhambazov, S. (2017). Cost-effectiveness assessment and budgetary implication of the Mamma print genetic test (70 genes) to determine the risk of relapse and the therapeutic strategy for the treatment of patients with early-stage breast cancer in Bulgaria, 2017 [Article]. General Medicine, 19(3), 34-40. | No full text available |
| Ramos, M. C. d. A., Folgueira, M. A. A. K., Maistro, S., Campolina, A. G., Soárez, P. C. d., Bock, G. H. d., Novaes, H. M. D., & Diz, M. D. P. E. (2018). Cost effectiveness of the cancer prevention program for carriers of the BRCA1/2 mutation. Revista de saude publica, 52. | No breast cancer. Mainly focused on ovarian cancer |
| Wei, X., Sun, H., Zhuang, J., Weng, X., Zheng, B., Lin, Q., Zhang, G., & Cai, J. (2020). Cost-effectiveness analysis of CYP2D6* 10 pharmacogenetic testing to guide the adjuvant endocrine therapy for postmenopausal women with estrogen receptor positive early breast cancer in China. Clinical Drug Investigation, 40(1), 25-32. | No BRCA1/2 testing |
| Bacchi, C. E., Prisco, F., Carvalho, F. M., Ojopi, E. B., & Saad, E. D. (2010). Potential economic impact of the 21-gene expression assay on the treatment of breast cancer in Brazil. Revista da Associacao Medica Brasileira, 56, 186-191. | No BRCA1/2 testing |
| Sánchez-Calderón, D., Pedraza, A., Mancera Urrego, C., Mejía-Mejía, A., Montealegre-Páez, A. L., & Perdomo, S. (2020). Analysis of the cost-effectiveness of liquid biopsy to determine treatment change in patients with her2-positive advanced breast cancer in Colombia. ClinicoEconomics and Outcomes Research, 115-122. | No BRCA1/2 testing |
| Aeilts, A. M., Carpenter, K. M., Hovick, S. R., Byrne, L., Shoben, A. B., & Senter, L. (2022). BRCAShare—Assessment of an animated digital message for intrafamilial communication of pathogenic variant positive test results: A feasibility study. Journal of Genetic Counseling. | No health economic evaluation |
| Frankenthal, I. A., Alves, M. C., Tak, C., & Achatz, M. I. (2022). Cancer surveillance for patients with Li-Fraumeni Syndrome in Brazil: A cost-effectiveness analysis. The Lancet Regional Health-Americas, 12, 100265. | No BRCA1/2 testing |
| Martínez del Prado, P., Alvarez-López, I., Domínguez-Fernández, S., Plazaola, A., Ibarrondo, O., Galve-Calvo, E., Ancizar-Lizarraga, N., Gutierrez-Toribio, M., Lahuerta-Martínez, A., & Mar, J. (2018). Clinical and economic impact of the 21-gene recurrence score assay in adjuvant therapy decision making in patients with early-stage breast cancer: pooled analysis in 4 Basque Country university hospitals. ClinicoEconomics and Outcomes Research, 189-199. | No BRCA1/2 testing |
| Grann, V. R., Patel, P. R., Jacobson, J. S., Warner, E., Heitjan, D. F., Ashby-Thompson, M., Hershman, D. L., & Neugut, A. I. (2011). Comparative effectiveness of screening and prevention strategies among BRCA1/2-affected mutation carriers. Breast cancer research and treatment, 125, 837-847. | No genetic testing evaluated |
| Özmen, V., Çakar, B., Gökmen, E., Özdoğan, M., Güler, N., Uras, C., Ok, E., Demircan, O., Işıkdoğan, A., & Saip, P. (2019). Cost effectiveness of gene expression profiling in patients with early-stage breast cancer in a middle-income country, Turkey: Results of a prospective multicenter study. European Journal of Breast Health, 15(3), 183. | No BRCA1/2 testing |
| Lipton, J. H., Zargar, M., Warner, E., Greenblatt, E. E., Lee, E., Chan, K. K., & Wong, W. W. (2020). Cost effectiveness of in vitro fertilisation and preimplantation genetic testing to prevent transmission of BRCA1/2 mutations. Human Reproduction, 35(2), 434-445. | Study from perspective of high income country |
| Pennarun, N., Chiu, J.-Y., Chang, H.-C., Huang, S.-L., & Cheng, S. H.-C. (2022). Cost-Effectiveness Analysis from a Societal Perspective of Recurrence Index for Distant Recurrence (RecurIndex) in Women with Hormone Receptor-Positive and HER2-Negative Early-Stage Breast Cancer. Cancer Management and Research, 761-773. | No BRCA1/2 testing |
| Oliveira, L. J. C., Megid, T. B. C., Rosa, D. D., Magliano, C. A. d. S., Assad, D. X., Argolo, D. F., Sanches, S. M., Testa, L., Bines, J., & Kaliks, R. (2022). Cost-effectiveness analysis of Oncotype DX from a Brazilian private medicine perspective: a GBECAM multicenter retrospective study. Therapeutic Advances in Medical Oncology, 14, 17588359221141760. | No BRCA1/2 testing |
| Bargalló-Rocha, J. E., Lara-Medina, F., Pérez-Sánchez, V., Vázquez-Romo, R., Villarreal-Garza, C., Martínez-Said, H., Shaw-Dulin, R. J., Mohar-Betancourt, A., Hunt, B., & Plun-Favreau, J. (2015). Cost-effectiveness of the 21-gene breast cancer assay in Mexico. Advances in therapy, 32, 239-253. | No BRCA1/2 testing |
| Katz, G., Romano, O., Foa, C., Vataire, A.-L., Chantelard, J.-V., Hervé, R., Barletta, H., Durieux, A., Martin, J.-P., & Salmon, R. (2015). Economic impact of gene expression profiling in patients with early-stage breast cancer in France. PloS one, 10(6), e0128880. | No BRCA1/2 testing |
| Nair, M. G., Ramesh, R. S., Naidu, C. M., Mavatkar, A. D., VP, S., Ramamurthy, V., Somashekaraiah, V. M., CE, A., Raghunathan, K., & Panigrahi, A. (2023). Estimation of ALU Repetitive Elements in Plasma as a Cost-Effective Liquid Biopsy Tool for Disease Prognosis in Breast Cancer. Cancers, 15(4), 1054. | No BRCA1/2 testing |
| Jacobs, V. R., Kates, R. E., Kantelhardt, E., Vetter, M., Wuerstlein, R., Fischer, T., Schmitt, M., Jaenicke, F., Untch, M., & Thomssen, C. (2013). Health economic impact of risk group selection according to ASCO-recommended biomarkers uPA/PAI-1 in node-negative primary breast cancer. Breast cancer research and treatment, 138, 839-850. | No BRCA1/2 testing |
| George, A., Riddell, D., Seal, S., Talukdar, S., Mahamdallie, S., Ruark, E., Cloke, V., Slade, I., Kemp, Z., & Gore, M. (2016). Implementing rapid, robust, cost-effective, patient-centred, routine genetic testing in ovarian cancer patients. Scientific reports, 6(1), 29506. | No breast cancer. Mainly focused on ovarian cancer |
| Kip, M., Monteban, H., & Steuten, L. (2015). Long-term cost–effectiveness of Oncotype DX® versus current clinical practice from a Dutch cost perspective. Journal of comparative effectiveness research, 4(5), 433-445. | No BRCA1/2 testing |
| Bacchi, C. E., Prisco, F., Carvalho, F. M., Ojopi, E. B., & Saad, E. D. (2010). Potential economic impact of the 21-gene expression assay on the treatment of breast cancer in Brazil. Revista da Associacao Medica Brasileira, 56, 186-191. | No BRCA1/2 testing |
| Quinn, V. F., Meiser, B., Kirk, J., Tucker, K. M., Watts, K. J., Rahman, B., Peate, M., Saunders, C., Geelhoed, E., & Gleeson, M. (2017). Streamlined genetic education is effective in preparing women newly diagnosed with breast cancer for decision making about treatment-focused genetic testing: a randomized controlled noninferiority trial. Genetics in Medicine, 19(4), 448-456. | No genetic testing evaluated |
| Wong, W. B., Ramsey, S. D., Barlow, W. E., Garrison Jr, L. P., & Veenstra, D. L. (2012). The value of comparative effectiveness research: projected return on investment of the RxPONDER trial (SWOG S1007). Contemporary clinical trials, 33(6), 1117-1123. | No BRCA1/2 testing |
